# Exploring Measures to Control Road Traffic Injuries in Iran: Key Informants Points of View

**Published:** 2017-05

**Authors:** Hedayat SALARI, Seyed Abbas MOTEVALIAN, Mohammad ARAB, Atefeh ESFANDIARI, Ali AKBARI SARI

**Affiliations:** 1. Dept. of Public Health, School of Public Health, Bushehr University of Medical Sciences, Bushehr, Iran; 2. Dept. of Health Economics and Management, School of Public Health, Tehran University of Medical Sciences, Tehran, Iran; 3. Dept. of Epidemiology, School of Public Health, Iran University of Medical Sciences, Tehran, Iran

**Keywords:** Measures, Control, Road traffic injuries, Iran

## Abstract

**Background:**

Injuries and fatalities from road traffic Injuries are global public health concerns, and a major problem in the Iran. This study aimed to explore strategies to control road traffic Injuries (RTI) in Iran.

**Methods:**

We conducted a qualitative study to explore possible ways to reduce the occurrence of road traffic Injuries in Iran in 2016. Interviewees were purposively sampled from various sectors due to multidisciplinary nature of RTIs. Participants were mainly representatives from the police, Ministry of Road, Municipal, emergency services and Ministry of Health. Besides, public health authorities, researchers, and university professors were interviewed. We conducted in-depth interviews using generic guides. Data was analyzed using MAXQDA 10 software. Through content analysis, we interpreted core themes relevant to the accomplishment of our study objectives.

**Results:**

Themes that emerged from our study include; road traffic management, governance, education, improving accident database, enforcement, driving license restrictions, and construction of pedestrian overpass.

**Conclusion:**

This study revealed key informants’ views regarding available and affordable solutions to reduce RTIs in Iran. Many applicable strategies are identified in the control of RTIs in Iran. Although some solutions such as highway construction and/or expanding rail transportation have been suggested as effective measures for reducing accident, but they are costly and may not be fully applied in developing countries like Iran.

## Introduction

Injuries and fatalities from road traffic Injuries are global public health concerns. Road traffic Injuries (RTIs) pose huge health and economic consequences to the Iranian society. RTIs remain one of the leading causes of death in the country. In 2009, more than 23000 deaths and 270000 injuries occurred in Iran were associated with RTIs. Besides, the cost of RTIs to the Iranian economy is approximately 64000 billion Rials – equivalent to 6.4% of the country’s GDP (1–3). In addition, much adverse health, social and economic consequences happened due to this devastating problem (2, 4).

Due to the burden of RTIs in Iran, measures to reduce their occurrence have gained special attention. Reducing deaths caused by traffic Injuries have become a major priority of the government and the health system (5, 6).

The annual death rate of RTIs was significantly high - about 30deaths per100000 in the past decades. Currently, RTIs deaths in Iran are considerably high compared with some developed countries, which have an average of 6 deaths per 100000 people (7, 8). Measure to reduce RT associated deaths have become a major priority of the government, policy makers and the health system (5, 6) since previous strategies did not yield expected result (a reduction from more than 30 deaths per 100000 to about 24 deaths per 100000). Unfortunately, people who annually lose their lives due to traffic Injuries throughout the country are more than the people who died due to the earthquake in Bam, Iran, in 2003.

According to high burden of injuries related to RTIs, the more attention of policymakers is necessary for applying better strategies. RTIs are major national crises and appropriate strategies should be put in place to reduce their occurrence. In view of these, we designed a research to explore the best possible solutions and/or appropriate strategies to reduce the burden of the problem in the country.

## Materials and Methods

We conducted a qualitative study to explore measures to address RTIs in 2016, using generic guides.Thirty interviewees were purposively sampled from various sectors due to its multidisciplinary nature of RTIs. Participants were mainly representatives from including; police, Ministry of road, Municipal, emergency services, Ministry of Health, with experience or knowledge in RTIs and its control mechanisms. In addition, researchers and university professors participated in the study. Interviewees were conducted until saturation was reached. Data was analyzed using MAXQDA 10 software. Through thorough content analysis, we interpreted core themes relevant to the accomplishment of our study objectives. Besides, two members (authors) performed code extraction.

This study was registered in the TUMS Ethical Committee (*Ref:* 9021460005). The participants’ permission to perform and audiotape the interviews was obtained. The confidentiality of information was guaranteed.

## Results

This study revealed seven main themes explaining measures to control RTIs in Iran. They include accident scene management, governance, education, improving accident database, enforcement of road traffic laws, driving license restrictions, and construction of pedestrians overpass (Table 1).

**Table 1:** Measures to control traffic Injuries in Iran

**Themes**	**Subthemes**
Accident scene management	Integration
	The use of a single Relay phone Number:
	Scientific examination of causes of accident
Governance and Leadership	Establishing a leading agency responsible for RTIs
Improving Accident database	Integrated Database
Education:	public education, and creation of awareness
Ensuring safe driving by Enforcement	random testing of the use of alcohol and drugs
	Increasing fines for traffic violations:
	Increasing the number of speed cameras
Ensuring safe driving by restriction	Instituting Psychological examination as part of the tests to acquire driver’s license
	Restricting teens from driving at night
Ensuring the safety of Pedestrians	Construction of pedestrian bridges/overpass:

### Accident scene management

#### Integration

There are several relay organizations that attend to casualties when an accident occurs However, according to study participants, these institutions perform separate duties without any cooperation, contributing to delays in rendering services to accident victims. As a result, participants suggested the need for an integrated functioning system that is well equipped to rescue accident victims.

“There should be only one well-equipped multipurpose team to rescue accident victims. This can increase the survival rates of casualties.”

(Top authority of Police)

#### The use of a single relay phone Number

Participants added that to have an integrated traffic relay system capable of delivering effective emergency services during a road accident, there should be a single communication system. Although in Iran, several phone numbers have already been registered for this purpose including 110, 125, 115, experts suggested that these different numbers should subscribe to a single [112] relay phone number. The callee should be in a position to perform several functions including giving immediate medical direction even before dispatching emergency teams to the accident scene.

“In my opinion, we need to a single rely on phone number like 112 in order to do timely and complete emergency services. Now there is deferent rely on phone numbers in Iran”(Expert)

#### Scientific examination of causes of accident

There should be scientific processes of determining causes of Injuries. “We should apply innovative and scientific techniques in managing RTIs. This can help us to identify the root cause of the accident, as to whether the occurrence was because of human error, poor transportation systems or inability of rescue teams due to delays or limited resources. Unfortunately, we [in Iran] do not have such systems.” (Top authority of EMS)

#### Governance and Leadership

Participants repeatedly emphasized the need for a vibrant leadership responsible for RTIs. According to them, as far as there is no sole organization to control RTIs, uncoordinated activities of numerous bodies cannot yield significant results. Although control of RTIs requires integrated measures, there should be a unified system with the sole authority and governmental support to control RTIs in Iran.

“There is not any real leading agency in charge of traffic injuries or a good governing system to steer the affairs of the issue. Besides, institutions do not have clear responsibilities to perform. They (institutions) do not have a unified working system that clearly defines the duties to address RTIs. This means that many organizations do their works separately, and their tasks are not transparent and well defined”. (Expert)

#### Knowledge and Public Awareness

There are low knowledge and awareness regarding the importance of complying with road safety measures such as the use of helmet, and the consequences of over-speeding. There should be interdisciplinary measure to increase awareness of and on adherence to road traffic laws. These they pinpointed can lead to a significant change in attitudes and risky behaviors of road users.

“People are ignorant of the dangers and consequences in disregarding road safety laws such as failure to wear a safety belt, and the need to maintain a significant distance away from moving motor vehicles or bikes when driving or walking. “.(Expert)

“Improving the quality of vehicles and construction of highways are expensive in terms of time and monies involved. We [Iran] should focus more on ways to change the behavior of road uses, especially drivers within the teen age. It should be considered in our educational systems” (Expert).

#### Improving Accident database

Valid information (data) is a prerequisite for effective control of RTIs and requires a database where information can be stored and shared among all stakeholders. “There is no definite statistics on injuries. There are several organizations currently collect data on traffic Injuries including Red Crescent, Ministry of Health, Forensic Medicine, police, insurance, etc. We can make the right decisions (scientifically proved) when we have good information systems and for that reason a good database. ”

### Enforcement

#### Random Alcohol and drug testing

Drivers should be randomly sampled to test for alcohol and drug use. This in their opinion can also contribute to curtailing RTIs in the country. “There is one thing that comes to mind; the use of drugs and alcohol. For example, about 50% of RTIs worldwide are attributed to alcohol consumption. Considering Iran traffic Injuries was the leading cause of death in the year 2004. Within the same period, abuse of opioids and disorder was ranked fifth. This is a problem because alcohol, drugs, and driving do not simply go together. Iran does not conduct random drug test unlike other European countries with considerably low rate of RTIs “(University professor).

#### Fines for traffic violations

One of the key strategies to reduce risky behaviors and violations of traffic laws by drivers is to impose and enforcing fines significant enough to deter people from such acts. Interviewees acknowledged that the current fees or fine are low (in Iran), and therefore should be increased in order to yield desired results.

“We should not compromise with people who violate the traffic law. Unfortunately, in our country [Iran] we do not want to increase the fines”(Expert).

#### Surveillance system

In order to reduce reckless driving, all roads should be fully covered by a network of fixed and mobile traffic speed cameras.

“One strategy to reduce traffic Injuries is fixing speed cameras on the roads. Speed cameras can reduce RTIs. We have cameras on the roads, but they are not enough” (Researcher).

### Driving license Restrictions

#### Psychological tests

Although issuing of driving license to the mentally ill is prohibited, psychological test does not form of certification process in Iran. People with mental disorders can also have access to a license to drive. In this respect, participants “Testing of mental (Psychological) abilities of candidates before issuing a driving license is a big issue in Iran which needs to be addressed. Measures have not been implemented to instill that in the country it is almost impossible to determine whether a person possessing a driving license is mentally sound to drive or not- I mean both private and commercial drivers. Such” (Road safety expert)

#### Restricting teens from driving at nights

Participants also raised the concern to restrict teens from driving at night. “Teenagers who succeed in getting a driver’s license should not be allowed to drive at night during their first year of driving.” (Road safety researcher)

#### Construction of pedestrian bridges/overpass

Every year a considerable number of pedestrians are being knockdown by vehicle, and one of the reasons according to our study participants is due to inadequate pedestrian overpass in the country. Although many pedestrian bridges have been constructed across the country, they are still not enough considering the population in the cities. They suggested that construction of sufficient pedestrian bridges across the country could prevent a larger percentage of RT injuries. Adding that, the construction of pedestrian bridges is a cost effective way to control RTIs, Besides, the bridges can be used for advertisement and can yield some income for the state.

“We should build more pedestrian bridges. Construction of pedestrian bridges can ensure the safety of our pedestrian. Besides, about 30 percent of people who die from road Injuries in Iran pedestrians?” (Road safety expert)

## Discussion

This study revealed the opinions of key informants’ regarding ways to reduce RTIs in Iran. Studies have explored several strategies to control traffic Injuries. Although certain recommendations, such as construction of highway and expansion of rail transportation have shown to be effective in addressing the phenomenon, they appear to be expensive especially for developing countries, and Iran is not an exemption.

A good governing system is important for effective control of RTIs and injuries. According to our findings, establishing a leading agency at the macro level is one of the most important strategies to control traffic Injuries. Despite the fact that many programs exist to reduce RTIs in the country, these programs have not achieved required outcome as anticipated due to the lack stewardship. There are numerous organizations responsible for the control of RTIs without a unified body having the full authority to steer the affairs of RTIs in the country. Automakers, road authorities, educators, the police, insurance companies, and the many other organizations responsible for prevention of traffic Injuries should have a leading organization responsible for tackling RTIs in an integrated manner (9, 10).

There are many institutions responsible for managing a disaster in Iran. Whenever there is a car accident, people close to the scene call various rescue team (numbers) such as 112, 115, 110 and 125, which leads to a delay in decision-making. One of the basic effective requirements to manage an accident or a disaster is the existence of a single emergency contact, capable of delivering quick and comprehensive services to casualties. A recent study conducted in Iran on post-crash management revealed lack of coordination between responsible bodies that resulted in delays conveying the victims to the health facilities. Moreover, our study participants pointed out the relevance of providing scientific reasons to why accidents occur. It is very important to apply scientific methods in identifying causes of accident least it is virtually difficult to prevent it from re-occurring (11).

Accurate information is an essential tool for good decision-making (12). The Islamic republic of Iran is faced with many challenges in controlling RTIs due to lack of information. There are inconsistencies in gathered data due to lack of coordination among the various organizations responsible for data collection. Besides, the Police do not use accurate tools in recording data due to lack of integrated database (13), and resources.

A wide range of interventions has been implemented worldwide to enforce preventive measures of RTIs and injuries. In this study, three themes related to enforcement were revealed including random alcohol and drug testing, increasing a number of fines for traffic violations, and increased the number of road/street cameras. It was emphasized by key informant’s opinion that drivers who do not adhere to laws should be severely punished. In Iran, the implementation of fines, have shown to be effective in controlling reckless behaviors and reducing RTIs and its associated burden.

Many pieces of evidence have revealed random alcohol and drug test to be useful in reducing RTIs. In addition, studies indicate the increase in fines and availability of speed cameras can control reckless driving and RTIs (14, 15). In our study, participants also added that early childhood education and creation of public awareness on RTIs could enhance the adherence of traffic law.

## Conclusion

The ability to drive is an important and a major requirement to secure driving license all over the world. Factors such as driver’s age and mental abilities should be considered before issuing a license to drive. Currently, the tests routinely performed during acquisition of driving license in Iran are physical examination and optometry. Teens usually drive recklessly at night, and thus restricting teens’ from driving at night will help minimize RTIs. Moreover, drivers should be psychologically sound, and more pedestrian overpass should be constructed to promote the safety of pedestrians (16).

## Ethical considerations

Ethical issues (Including plagiarism, informed consent, misconduct, data fabrication and/or falsification, double publication and/or submission, redundancy, etc.) have been completely observed by the authors.
